# Editorial: Fibrotic tissue remodeling as a driver of disease pathogenesis

**DOI:** 10.3389/fmolb.2023.1278388

**Published:** 2023-08-29

**Authors:** Trayambak Basak, Sarika Saraswati

**Affiliations:** ^1^ School of Biosciences and Bioengineering, Indian Institute Of Technology–Mandi (IIT–Mandi), Mandi, Himachal Pradesh, India; ^2^ BioX Center, Indian Institute Of Technology–Mandi (IIT–Mandi), Mandi, Himachal Pradesh, India; ^3^ Department of Biological Sciences, Tennessee State University, Nashville, TN, United States

**Keywords:** cardiac fibrosis, extracellular matrix (ECM), regeneration, scar, healing

Fibrosis has emerged as one of the main pathophysiological common end-point in organ failure. Maladaptive tissue remodeling and fibrosis are typical responses to injury which leads to distorted architecture, pathologic signaling, and extracellular matrix (ECM) remodeling leading ultimately to organ dysfunction (Henderson et al., 2020). Tissue fibrosis has remained the main pathophysiological endpoint outcome leading to organ failure causing severe morbidity worldwide (Bollong et al., 2017). Cardiac fibrosis leading to heart failure and other forms of cardiovascular diseases (CVD) is a major contributor to global mortality (Gulati et al., 2013). Clinically, patients suffering from CVDs are often attributed to cardio-renal and cardio-pulmonary complexities as well (Husain-Syed et al., 2015). A histological hallmark of many of these end-stage pathologies is tissue fibrosis, characterized by an excessive accumulation of extracellular matrix (ECM) molecules and de-regulation of ECM modifying enzymes (Lu et al., 2011; Bollong et al., 2017). The cause-consequence relation between this fibrotic remodeling and the patient’s outcome remains unclear—and likely depends on the type of tissue insult. Further, perturbed cellular signaling could lead to myofibroblast activation contributing to inherent fibrosis development (Duffield, 2014; Gibb et al., 2020). The degree of tissue fibrosis, comorbidities, and the standard of care therapies often vary among patients (Raghu et al., 2015; Wong et al., 2020). Further, the limited availability of effective anti-fibrotic therapies has remained a major bottleneck in the reversal of the tissue fibrosis prognosis. Therefore, effective, safe, and well-tolerated pharmacological strategies that halt fibrotic remodeling of tissues are an area of unmet but high clinical need. The challenges are to prospectively define patient populations that benefit from anti-fibrotic therapies and gain a deeper mechanistic understanding of pro-fibrotic signaling mechanisms. These molecular mechanistic understandings eventually would lay the foundation for developing next-generation therapeutics to tackle pathological fibrosis in a spatio-temporal manner.

In this Research Topic, our focus was to highlight studies that primarily addressed the factors involved in modulating fibrosis in cardiac repair as well as other tissue pathologies including chronic kidney diseases, keloids, type II diabetes and glaucoma. Both Drs. Haffner and Leifheit-Nestler’s groups have indicated the significance of FGF23 in cardiac and kidney pathologies (Leifheit-Nestler et al.; Eitner et al.). Dr. Haffner’s group identified that FGF23 expression increased in cardiac fibroblasts and endothelial cells following pressure-induced cardiac hypertrophy and not in cardiac myocytes (Eitner et al.). However, Cre-Flox mediated deletion of FGF23 in myocytes resulted in worsening of cardiac function followed by transverse aortic constriction (TAC) induced cardiac insult (Eitner et al.). The protective role of FGF23 on cardiac function was also demonstrated by Dr. Leifheit-Nestler’s group by utilization of adeno-associated virus (AAV) expressing murine Fgf23 system. They demonstrated that even though FGF23 is elevated in chronic kidney disease (CKD) and is associated with left ventricular hypertrophy (LVH) in CKD, it is not cardiotoxic and does not induce LVH in healthy mice (Leifheit-Nestler et al.). These studies emphasized the fact that increased expression of a protein does not always translates into its pathological role in tissue pathology. Increased expression of FGF23 could be a protective compensatory mechanism to tackle the insult (Eitner et al.).

Using Zebrafish as a model, Dr. Basak’s group teased out the mechanism of cardiac ECM remodeling. Collagen molecules are the major players in post-injury cardiac remodeling. Additionally, post-translational modifications including hydroxylation of proline, lysine, and glycosylation of lysine have been shown to be critical in modulating physiological as well as disease-related extracellular-matrix interaction with its cellular microenvironment (Sarohi et al.). This is the first comprehensive study to perform site-specific collagen post-translational modification map of zebrafish heart. Since zebrafish have a unique capability to regenerate the heart following injury, this study will be instrumental in decoding extracellular-matrix remodeling and identifying potential therapeutic targets for the mammalian post-injury fibrotic response.

Fibrosis is not an organ-specific response. It is a physiological as well as pathological response following any kind of tissue injury as well pathological condition. Keloids is a fibroproliferative cutaneous disease with limited diagnostic indicators for early screening and prevention. Xia et al. performed an extensive evaluation of the Gene Expression Omnibus database of keloid matrix files to identify keloid-specific gene signatures and correlated it with immune cell infiltration. Transglutaminase 2 was identified as a significant indicator for keloids and may serve as a diagnostic as well as a therapeutic tool for future studies (Xia et al.). Complementing this study, Dr. Xu’s group emphasized the role of circular RNA in keloid pathophysiology and identified hsa_circ_0006867 as a biomarker of keloids (Pang et al.).

Another interesting study published in the Research Topic was the gene ontology analyses of human islets derived from type 2 diabetes mellitus patients. In addition to high glucose-induced activation of extracellular matrix pro-fibrotic proteins, Lu et al. identified IL6 and IL11 as key inflammatory cytokines that may promote fibrosis-induced islet dysfunction (Lu et al.). This *in silico* analysis was confirmed in an obese rat animal model. The effect of high glucose levels in inducing inflammation and subsequently fibrosis and islet dysfunction is an important avenue of research and should get more attention in diabetes research.

Fibrotic activity in the human trabecular meshwork (HTM) cells can be one major factor that promotes high intraocular pressure leading to glaucoma (Liu et al.). Through bioinformatics supported by mechanistic studies, Liu et al. identified RhoA/ROCK-YAP/TAZ axis as having a crucial role in modulating the fibrotic activity of dexamethasone-treated human trabecular meshwork cells (Liu et al.).

Tissue fibrosis is a complex process that involves the interplay between multiple cell types including fibroblasts, immune cells, and endothelial cells. Fibrosis accompanies wound healing where it drives the failure of many organs. However, post-injury repair through fibrosis also ensures organ integrity making it harder to target. Understanding the molecular mechanisms regulating post-injury fibrotic healing is crucial in addressing unchecked fibrosis without interfering with the healing process. After decades of research, fibrosis research is still in its infancy to understand this inevitable pathological post-injury outcome. In this Research Topic, we have published some molecular targets in a wide range of tissues that should be further investigated and may provide a significant clue in tipping the balance towards regenerative healing instead of fibrosis.
